# Hypoglossal Nerve Palsy Following COVID-19 Vaccination in a Young Adult Complicated by Various Medicines

**DOI:** 10.7759/cureus.29212

**Published:** 2022-09-15

**Authors:** Tatsuhiko Okayasu, Ryuichi Ohta, Fumiko Yamane, Satoshi Abe, Chiaki Sano

**Affiliations:** 1 Family Medicine, International University of Health and Welfare, Tokyo, JPN; 2 Community Care, Unnan City Hospital, Shimane, JPN; 3 Neurology, Shimane Medical University Hospital, Izumo, JPN; 4 Community Medicine Management, Shimane Medical University Hospital, Izumo, JPN

**Keywords:** general medicine, rural hospital, extremity paralysis, immunoglobulin infusion, steroid, hypoglossal nerve palsy, mononeuritis multiplex, covid-19 vaccination

## Abstract

Mononeuritis multiplex is a rare form of cerebral nerve palsy caused by various factors. Coronavirus disease 2019 (COVID-19) vaccination could be an etiology of mononeuritis multiplex, which can affect various nerves. Post-COVID-19 and vaccination-related neurological impairments involve cranial nerves such as the facial, trigeminal, and vagal nerves. We report our experience with a 34-year-old man who developed hypoglossal nerve palsy following COVID-19 vaccination, complicated by progressive mononeuritis multiplex. Hypoglossal nerve palsy may occur following COVID-19 vaccination. The symptoms vary and may progress without treatment. Physicians should consider the possibility of mononeuritis multiplex after COVID-19 vaccination and provide prompt treatment for acute symptom progression.

## Introduction

Mononeuritis multiplex is a rare form of cerebral nerve palsy caused by various factors, as the etiologies, infection, and autoimmunity are common. Herpes zoster and simplex are the predominant infections in the category of infection [1,2]. Among autoimmune causes, small-to-medium-sized vasculitis, such as an antineutrophil cytoplasmic antibody (ANCA)-related vasculitis and Sjogren’s syndrome, are common [1,2]. The progression of mononeuritis multiplex symptoms varies depending on the human body's etiology and immunological reactions [3,4]. Severe cases may involve multi-extremity paralysis, which should be treated with intravenous immunoglobulin therapy, steroids, and plasma exchange, according to the etiology [2,5]. Thus, effective treatment requires the detection of etiology and rapid treatment.

COVID-19 and COVID-19 vaccinations are also potential etiologies of mononeuritis multiplex, which can affect various nerves. Based on previous reports, post-COVID-19 and vaccination-related neurological impairments involve cranial nerves such as the facial, trigeminal, and vagus nerves [6-8]. However, there are few reports of mononeuritis multiplex following COVID-19 vaccination. Here, we report a case of mononeuritis multiplex that spread from the right hypoglossal nerve to the right hand and leg. The progression was acute, and the patient required treatment with intravenous immunoglobulin and steroid pulse therapy. Various complications occurred during the clinical course, and the treatment course was complicated. Our case demonstrates the importance of a clinical diagnosis of mononeuritis multiplex with prompt treatment and approaches to reduce long-term complications.

## Case presentation

A 34-year-old man was admitted to our hospital with a chief complaint of dysphasia and difficulty speaking. Ten days before admission, the patient had received the third vaccination for COVID-19. He had a fever of >38 °C one day after vaccination. Seven days before admission, he experienced tingling on the right side of his tongue, followed by dysphagia and difficulty speaking. These symptoms progressed, and the patient noticed that the right side of his tongue had shrunk; therefore, he visited our hospital. He had a past medical history of varicella-zoster virus infection in the first branch of the left trigeminal nerve and had been treated with valaciclovir. The patient did not take any regular medication.

**Figure 1:**
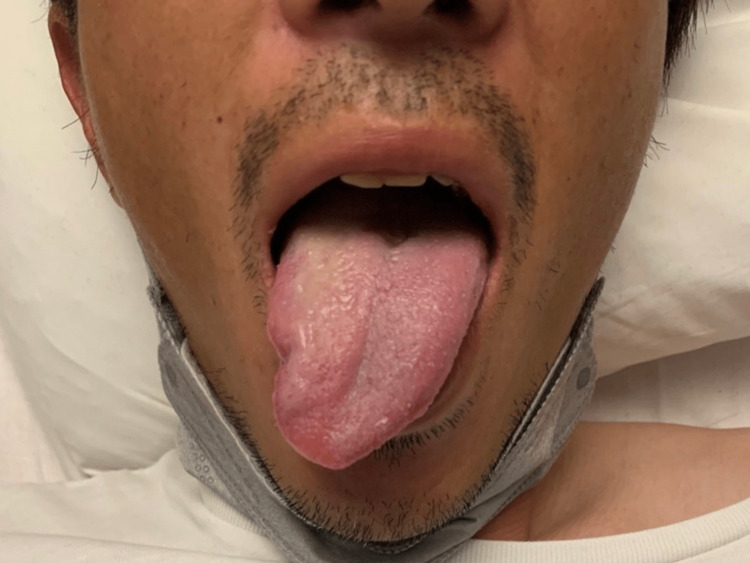
Right sublingual neurological paralysis

His vital signs at admission were as follows: blood pressure, 114/59 mmHg; pulse rate, 78 beats/min; body temperature, 36.9 °C, respiratory rate, 15 breaths/min; and oxygen saturation, 97% on room air. He was alert to time and place. Physical examination showed that the right half of his tongue was atrophied and shifted to the right during the prostration (Figure 1).

No other abnormal neurological findings were noted. There were no obvious abnormalities in the chest or abdomen and no skin eruptions. Physical examination revealed right hypoglossal nerve palsy; thus, viral infection, brain stroke, brain tumor, meningitis, ANCA-related vasculitis, and Guillain-Barre syndrome was suspected. Blood tests, head magnetic resonance imaging (MRI), head computed tomography (CT), and lumbar puncture were performed. The results were within normal limits (Table 1).

**Table 1:** Initial laboratory data of the patient eGFR: estimated glomerular filtration rate; CK: creatine kinase; CRP: C-reactive protein

Marker	Level	Reference
White blood cells	6.8	3.5–9.1 × 10^3^/μL
Neutrophils	51	44.0–72.0%
Lymphocytes	32.9	18.0–59.0%
Monocytes	8	0.0–12.0%
Eosinophils	6.9	0.0–10.0%
Basophils	1.2	0.0–3.0%
Red blood cells	5.34	3.76–5.50 × 10^6^/μL
Hemoglobin	16	11.3–15.2 g/dL
Hematocrit	47.8	33.4–44.9%
Mean corpuscular volume	89.5	79.0–100.0 fl
Platelets	24.6	13.0–36.9 × 10^4^/μL
Total protein	6.9	6.5–8.3 g/dL
Albumin	4.4	3.8–5.3 g/dL
Total bilirubin	0.5	0.2–1.2 mg/dL
Aspartate aminotransferase	18	8–38 IU/L
Alanine aminotransferase	27	4–43 IU/L
Alkaline phosphatase	80	106–322 U/L
γ-Glutamyl transpeptidase	50	<48 IU/L
Lactate dehydrogenase	165	121–245 U/L
Blood urea nitrogen	13.9	8–20 mg/dL
Creatinine	0.66	0.40–1.10 mg/dL
eGFR	≥90	> 60.0 mL/min/1.73 m^2^
Serum Na	137	135–150 mEq/L
Serum K	3.9	3.5–5.3 mEq/L
Serum Cl	101	98–110 mEq/L
Serum P	3.1	2.7–4.6 mg/dL
Serum Mg	2	1.8–2.3 mg/dL
CK	112	56–244 U/L
CRP	0.07	<0.30 mg/dL
Artery blood gas analysis		
pH	7.418	7.35–7.45
PCO_2_	42.5	35.0–45.0 mmHg
PO_2_	89.3	75.0–100.0 mmHg
HCO_3_	26.9	20.0–26.0 mmol/L
Lactate	1.2	0.5–1.6 mmol/L
Cerebrospinal fluid testing		
Color	clear	
Cell count	1	0–5 /μL
Protein	36	15–45 mg/dL
Glucose	57	48–83 mg/dL
Chloride	126.5	113–128 mEq/L

A videoendoscopic examination of swallowing was performed to evaluate dysphagia, with no obvious problems associated with swallowing function. Since the difficulty in moving the tongue and the white coating was remarkable, the patient was referred to a dental and oral surgeon to rule out tongue cancer.

**Figure 2:**
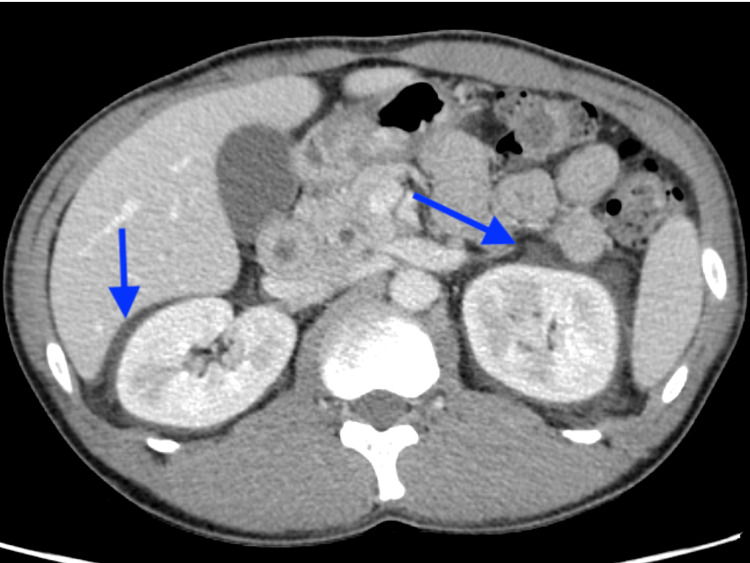
Edematous findings around both kidneys (blue arrows)

Because the patient had a history of herpes zoster, we also considered viral reactivation and prescribed acyclovir (1500 mg/day) and prednisolone (60 mg/day) from the second day of admission. However, lumbar pain and headache appeared on day four of admission, for which epidural hematoma after lumbar puncture was suspected. Plain lumbar magnetic MRI and head CT showed edematous findings around both kidneys, clinically suggesting the possibility of acute kidney injury due to acyclovir. As the patient tested negative for varicella virus, acyclovir was discontinued (Figure 2).

On the seventh day of illness, weakness of the right upper and lower extremities and a Romberg's sign was observed. Plain MRI of the upper arm and nerve conduction velocity tests were performed to investigate the cause, with no positive findings. Blood tests were negative for syphilis, hepatitis, HIV, ANCA, antinuclear antibody, and IgG4. Therefore, a clinical diagnosis of mononeuritis multiplex after administering the COVID-19 vaccine was made. On day seven of admission, prednisolone (60 mg/day), intravenous immunoglobulin (0.4 g/kg/day for five days), and methylprednisolone (1 g/day for three days) were initiated after consultation with a neurology physician. On day nine of admission, muscle pain, and general malaise developed immediately after intravenous methylprednisolone administration. As intravenous methylprednisolone could be the cause, the administration was discontinued, and oral prednisolone (60 mg/day) was started. Subsequently, a tingling pain appeared on the right scalp. He was treated with valacyclovir (3 g/day for one week). Dysphagia and extremity weakness gradually improved after rehabilitation. On day 14, after admission, the patient was transferred to a university hospital for further investigation and advanced rehabilitation.

## Discussion

This case showed the possibility of hypoglossal nerve palsy as a rare complication of COVID-19 vaccination, specific neurological complications following COVID-19 vaccination, and the rapid treatment of mononeuritis multiplex to prevent symptom progression.

The relationship between the COVID-19 vaccine and mononeuritis multiplex has been discussed in various studies. Several case reports have shown an increased risk of mononeuritis multiplex within a few days to months after COVID-19 vaccination [8,9]. A review of COVID-19 vaccination also showed that most symptoms related to mononeuritis multiplex were mild and disappeared naturally [10]. However, some cases show severe symptoms that affect the patient’s activities of daily life and require intensive treatment [7,11]. Our patient initially had mild symptoms and did not require treatment for his vital symptoms. However, within one week, the symptoms progressed drastically from the tongue to the extremities, causing difficulties in walking. The clinical course of mononeuritis multiplex varies, and some cases caused by vasculitis from autoimmune and infectious diseases may be progressive [5,12]. Precise follow-up and prompt treatment with intravenous immunoglobulins and steroids should be initiated to prevent disease progression.

Hypoglossal nerve palsy could be a rare symptom following COVID-19 vaccination and warrants further investigation in future studies. Among the complications of COVID-19 vaccination, various neurological complications were reported in 2020 [9,10]. Guillain-Barre syndrome is a well-known but rare complication of COVID-19 vaccination and appears a few weeks after vaccination [13]. Other cranial nerves may also be involved in the complications of COVID-19. Several case reports and reviews have reported facial palsy, the pain of the trigeminal and facial nerves, and diplopia of the oculomotor nerves [10,14]. However, hypoglossal nerve palsy is rare, and its pathophysiology remains unclear. In the present case, the initial finding was difficulty in tongue movement caused by palsy of the hypoglossal nerves, which led to systemic neurological symptoms. Clinicians should consider assessing single cranial symptoms following COVID-19 because of the possible spread of multiple nerve symptoms, causing a decreased quality of life.

The COVID-19 pandemic may persist in the future; therefore, preventable measures are vital. Vaccination is a critical measure for prevention. Although various complications have been reported, they are rare; therefore, vaccination should be promoted [15,16]. However, the possible symptoms following COVID-19 vaccination should be appropriately described, and help-seeking behaviors (HSB) to medical facilities should be facilitated, especially in rural contexts lacking healthcare resources [17-19]. The patient in the present case was younger, but the duration of his visit to the hospital was nearly two weeks. Early treatment could have prevented symptom progression [14]. When the same symptoms occur in older patients, HSB varies and is challenging, causing a greater delay in treatment. Citizens and healthcare professionals should be educated regarding responses to symptoms following vaccination, and information provision should be promoted [20].

## Conclusions

Hypoglossal nerve palsy may be a symptom of COVID-19 vaccination. The symptoms vary and may progress without treatment. Physicians should consider the possibility of mononeuritis multiplex after COVID-19 vaccination and provide prompt treatment for acute symptom progression.
